# A meta-ethnography exploring parents’ experiences of fetal death and the care they received between diagnosis and birth induction

**DOI:** 10.18332/ejm/200614

**Published:** 2025-03-19

**Authors:** Cathrine H. Pettersson, Tone H. Sand, Bente Dahl

**Affiliations:** 1 Faculty of Health Sciences, University of Stavanger, Stavanger, Norway

**Keywords:** communication, qualitative, midwifery care, meta-ethnography, fetal death, parents’ experiences

## Abstract

**INTRODUCTION:**

Being notified that the fetus has died *in utero* is an extremely challenging situation for parents, involving emotional chaos, shock, and despair. Healthcare professionals also find it challenging to cope with these situations. The purpose of this study was to explore parents' experiences of fetal death and the care they received between diagnosis and birth induction.

**METHODS:**

We conducted a meta-ethnography in accordance with Noblit and Hare’s seven phases and the eMERGe reporting guidance. Literature searches were conducted in CINAHL, MEDLINE, PubMed and PsycInfo in October and November 2022 and updated in August 2024. A PRISMA flowchart was used to illustrate the search process and quality assessment was performed according to CASP.

**RESULTS:**

Seven studies were included in the meta-ethnography. ‘Realizing the unreal by taking control of the uncontrollable’ emerged as an overarching metaphor through a reciprocal translation of the data. The metaphor illustrated four main themes: 1) Falling into the unknown, 2) Needing care during vulnerable times, 3) Communicating the meaningless, and 4) Navigating the terrain. Receiving the diagnosis resulted in feelings of chaos. However, healthcare providers possessing empathetic communication skills helped them to cope with the situation and prepare for birth.

**CONCLUSIONS:**

Optimal care performance, where communication is central, is a useful strategy for healthcare professionals in their encounters with parents who experience fetal death. Training is vital in order to provide good care and support for parents throughout the process.

## INTRODUCTION

A full-term birth is considered to occur between gestational weeks 38 and 42. When the fetus dies in the womb, this is referred to as early fetal death if it occurs after 22 weeks of gestation or when the fetus weighs >500 g. After 28 weeks of gestation or when the fetus weighs >1000 g, this is referred to as late fetal death. According to the International Classification of Diseases 10th revision (ICD-10), the term ‘stillbirth’ is used to refer to all early and late fetal deaths^1^. However, a variability in definitions has developed over time, and the ready availability of intensive care interventions that has resulted in increased viability has influenced the definition^2^. Fetal death is usually diagnosed *in utero* by the absence of fetal heart sounds and is confirmed by ultrasound or the absence of signs of life at birth, or after attempts at resuscitation^1^. Placental pathology, infections, umbilical cord complications, maternal and fetal pathologies, congenital malformations, and intrapartum pathology are the primary causes of fetal death, but despite an autopsy the cause of death in 20–30% of cases remains unknown^3^.

Previous research has demonstrated that local and national guidelines mainly address the practical aspects rather than the emotional aspects of caring for parents who experience fetal death^4^. Nevertheless, losing a child has many psychosocial and existential issues for a family, involving shock, panic, hope, despair, and increased parental mortality^5,6^. This is a severe traumatic event, and these parents require proper support and respectful attitudes, and care from healthcare professionals (HCPs)^5^. Nevertheless, research has shown great variation in the care that grieving parents experience and emphasizes the inadequate levels of communication between HCPs and parents^7^. Information is often characterized by uncertainty and is difficult for parents to comprehend^5^. Although fetal death occurs regularly in clinical practice, midwives and other HCPs still find it challenging to cope with these situations^8,9^. The incident is likely to affect midwives emotionally, and being part of the event is often perceived as unsafe, painful, and frightening^10^. Research also shows that midwives have not been adequately trained in providing care for parents in the event of fetal death, neither during their education nor in clinical practice^10^.

Being notified that the fetus has died *in utero* is an extremely challenging situation for parents, involving emotional chaos, shock, and despair. However, few qualitative studies have focused primarily on parents’ experiences of fetal death in the period between diagnosis and the induction of birth^11^. Recent research highlights the need to explore the emotional support and care that parents receive from midwives and other HCPs^12^ in this situation. Identifying the focus of the study and defining the study aim constitutes the first phase in meta-ethnography, i.e. ‘Getting started’^13^. The purpose of this meta-ethnography was to synthesize qualitative research exploring parents’ experiences of fetal death and the care they received between diagnosis and birth induction. The scarcity of qualitative research in this field emphasizes the complexity of parents’ emotional experiences related to fetal death. Thus, meta-ethnography was a suitable means to provide valuable insight on the topic. Studies describing parents’ experiences of fetal death can help midwives better understand how the situation is perceived by the parents, allowing healthcare professionals, particularly midwives, to improve care during this critical period, by synthesizing a broader exploration of parents’ experiences and emotional aspects. The research question was: ‘What are parents’ experiences of fetal death and the care they received between diagnosis and birth induction?’.

## METHODS

Meta-ethnography is an interpretative methodology for qualitative evidence synthesis, drawing on the Geertz^14^ concept of ‘thick descriptions’ (implying deep interpretations, contextualizing and implications of the phenomenon) as well as Turner’s theory of sociological understanding as ‘translation’^15^ (referring to the importance of interpreting and translating symbols, rituals, dramas and understanding to gain deeper knowledge of society and its structures).

We conducted the study according to the Noblit and Hare^13^ description of meta-ethnography, consisting of seven overlapping and iterative phases shown in Table 1. Below, we provide a description of our work according to these phases. We also used the eMERGe reporting guidance^16^ to ensure open and complete reporting of the meta-ethnography (Supplementary file Table 1). The eMERGe reporting guidance includes 19 reporting criteria, covering the seven phases of meta-ethnography.

**Table 1:** Phases in meta-ethnography^13^

*Phases*	*Strategies*
1. Getting started	Identifying the topic of the study and defining the aim.
2. Deciding what is relevant to the initial interest	Including relevant studies, describing search strategy and criteria for inclusion and exclusion.
3. Reading the studies	Noting studies’ interpretative metaphors through repeated readings.
4. Determining how the studies are related	Determining the relationship between the studies by creating a list of key metaphors (themes, concepts, phrases, ideas) and assessing whether the relationships are reciprocal (i.e. findings across studies are comparable), refutational (findings stand in opposition to each other) or represent a line of argument.
5. Translating the studies into one another	Comparing metaphors and their interactions within single studies and across studies, and at the same time protecting uniqueness and holism.
6. Synthesizing translations	Creating a new whole from the sum of the part, enabling a second-level analysis.
7. Expressing the synthesis	Finding the appropriate form for the synthesis to be effectively communicated to the audience.

### Search strategy and search processes

In the second phase, ‘Deciding what is relevant to the initial interest’, we conducted a literature search. The search strategy was shaped by the research aim and the purpose of the meta-ethnography to capture qualitative research focused on parents’ emotional, experiential, and care-related aspects in the context of fetal death. A scoping search was conducted in May and June 2022 in the CINAHL, MEDLINE, and PubMed databases. This was followed by a revision of the search terms before systematic literature searches were conducted in October and November 2022 and updated in August 2024. The following databases were used: CINAHL, MEDLINE, PubMed, and PsycINFO. The SPIDER system was used^17^, which is a well-suited and appropriate tool and includes: (S) selection, (PI) phenomenon of interest, (D) design, (E) evaluation, and (R) type of research. The search terms used are described in Supplementary file Table 2. We used Boolean operators to combine the terms as follows: (S) AND (PI) AND (E) AND (D OR R). Truncations were used to ensure a sufficiently broad search. Finally, we conducted a manual search, backtracking the reference lists in the included articles. All authors were responsible for carrying out the literature searches, and a librarian assisted in identifying relevant key terms and performing the searches.

In the first step, two authors (CHP, THS) read the titles of the articles and irrelevant publications were removed. In the next step, we reviewed abstracts and excluded irrelevant articles or articles using unsuitable methodologies. Finally, we read through the articles for relevance and quality, and performed a critical assessment of the articles.

### Primary studies selection

**Figure 1:**
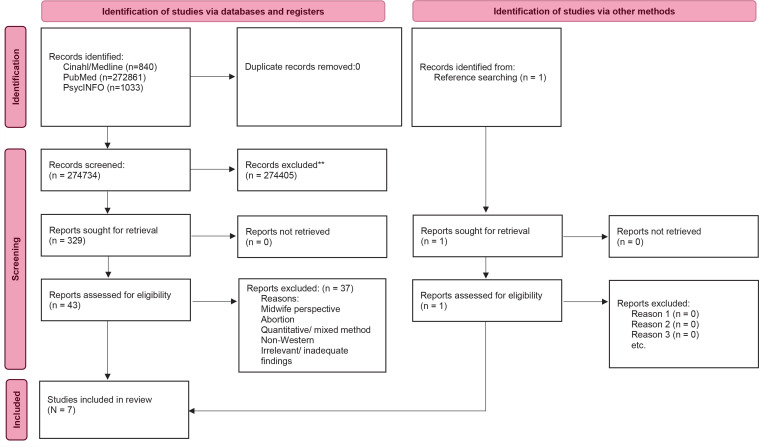
PRISMA flow diagram for selection of studies

Two authors (CHP, THS) were involved in the screening process, using a PRISMA^18^ flow diagram (Figure 1). The process started with individual readings and was followed by discussions with all the authors to reach a consensus on eligibility and inclusion. We included qualitative, peer-reviewed empirical articles written in English or Scandinavian languages and published in scientific journals between 2012 and 2024. The articles had to focus on parents’ experiences of fetal death after pregnancy week 22, including information about their experiences with the care they received between the diagnosis and the birth induction.

We included studies conducted in high-income contexts because we were interested in maternity care systems that could be compared to the Norwegian system. We included research irrespective of maternal parity, time since death, and cause of death. We excluded articles dealing with abortion and studies describing midwife or other HCP perspectives. We also excluded quantitative articles and articles using mixed methods, theoretical papers, reviews, Master’s theses, and dissertations.

### Quality appraisal

Two authors critically assessed the included articles using a checklist from the Critical Appraisal Skills Programme (CASP)^19^. The checklist consists of 10 questions. It does not offer a scoring system but is a pedagogical tool that serves as a systematic reminder of study quality. Articles eligible for inclusion were divided between two of the authors (CHP, THS) and assessed independently. When minor disagreements occurred, they were discussed in the research team and a final consensus was reached. The quality appraisal is described in detail in Table 2.

**Table 2:** Quality assessment of included studies (CASP)^19^

*Authors*	*Year*	Assessment questions*									
		*1*	*2*	*3*	*4*	*5*	*6*	*7*	*8*	*9*	*10*
Erlandsson et al.^11^	2011	Y	Y	Y	Y	Y	Y	Y	Y	Y	Y
Trulsson and Rådestad^21^	2004	Y	Y	C	Y	Y	Y	Y	Y	Y	Y
Martínez-Serrano et al.^22^	2019	Y	Y	Y	Y	Y	Y	Y	Y	Y	Y
Rådestad et al.^23^	2014	Y	Y	Y	Y	Y	Y	Y	Y	Y	Y
Malm et al.^24^	2011	Y	Y	Y	Y	Y	Y	Y	Y	Y	Y
Downe et al.^25^	2013	Y	Y	C	Y	Y	Y	Y	Y	Y	Y
Camacho-Ávila et al.^26^	2019	Y	Y	Y	Y	Y	Y	Y	Y	Y	Y

Y: yes. N: no. C: cannot tell.

* Assessment questions: 1) Was there a clear statement of the aims of the research?; 2) Is a qualitative methodology appropriate?; 3) Was the research design appropriate to address the aims of the research?; 4) Was the recruitment strategy appropriate to the aims of the research?; 5) Were the data collected in a way that addressed the research issue?; 6) Has the relationship between researcher and participants been adequately considered?; 7) Have ethical issues been taken into consideration?; 8) Was the data analysis sufficiently rigorous?; 9) Is there a clear statement of findings?; and 10) How valuable is the research?

### Reading and data extraction approach

Before starting the analysis, a critical reading of the selected articles was carried out by two authors (CHP, THS). We focused on identifying metaphors (themes, categories, concepts, phrases, citations) that could help illuminate the study aim, according to the third phase ‘Reading the studies’. We developed a table describing the study aim, sample characteristics, research design, data collection, data analysis, and key findings of the included studies in order to provide context (Table 3).

**Table 3:** Characteristics of included studies

*Authors Year Country*	*Aim*	*Research design*	*Sample and setting*	*Methods for data collection and analysis*	*Key findings*
Erlandsson et al.^11^ 2011 Sweden	To investigate how mothers allocated the time from diagnosis of fetal death was set up to induction.	Qualitative design	515 mothers	Online questionnaire Qualitative content analysis	Some mothers received help to adapt to the situation in anticipation of induction, while others experienced stress and psychological trauma as an extension of an already strained situation.
Trulsson and Rådestad^21^ 2004 Norway	To investigate why the time between diagnosis of fetal death and induction of labor for most mothers should not be longer than 24 hours, and how the time should be allocated.	Qualitative design	12 mothers	Interview Phenomenological method	Mothers experienced a feeling that something was wrong and had difficulty communicating their concerns. They felt that verbal communication with staff ceased, that the situation was unreal and that they felt numbness. Some mothers had an immediate need to get rid of the dead child and the total silence. Several mothers felt that they were not respected at the time of diagnosis.
Martínez-Serrano et al.^22^ 2019 Spain	To investigate mothers’ and fathers’ experiences of the care they received during stillbirth.	Qualitative design	7 mothers 4 fathers	Semi-structured interviews Inductive thematic analysis Hermeneutic phenomenology	Four main themes were identified: ‘Denial of grief’, ‘The life and death paradox’, ‘Guilt’ and ‘Going through and overcoming the loss’. The parents experienced a lack of recognition of the loss and of their parenting role. The midwife was the one who was most valued, but not all the experiences were good, and the parents had appreciated healthcare personnel with good communication skills. Circumstances at the hospital complicated the process and the focus was on the birth taking place in the same place where live children were to be born.
Rådestad et al.^23^ 2014 Sweden	To investigate mothers’ experiences of receiving notification with confirmation of fetal death on ultrasound.	Qualitative design	26 mothers	In-depth interview Qualitative inductive content analysis	During the ultrasound, the mothers felt that silence reigned. The health personnel involved were focused and concentrated, no one spoke to them. Based on the mothers’ observations on the ultrasound screen and how they interpreted the body language of the healthcare providers involved, their instincts suggested that the baby could be dead. Some mothers experienced uncertainty and time delays in receiving information about their baby’s death.
Malm et al.^24^ 2011 Sweden	To investigate the mothers’ experiences of the time from diagnosis of fetal death to the start of labor.	Qualitative design	21 mothers	In-depth interview Qualitative inductive content analysis	‘Waiting in no man’s land’ emerged as an overarching theme based on the mothers’ experiences. The theme describes going from normal and familiar existence into an unfamiliar situation. Four categories were identified: ‘Involuntary waiting’, which is about being left without information about what is to come; ‘Dealing with the unimaginable’, being in a confusing and terrible situation and yet having to deal with childbirth; ‘Broken expectations’, is about the loss of the baby, but also of the future family life they envisioned; and ‘Courage to face life’, which implied the willingness to persevere and face reality.
Downe et al.^25^ 2013 UK	To examine bereaved parents’ views on the relationship they had with healthcare professionals when their baby died before or during birth.	Qualitative design	25 parents	Online questionnaire followed by in-depth interviews, face-to-face or over the phone Grounded theory	The relationship with healthcare personnel had a major impact on the parents’ ability to cope with childbirth and the time after. The overarching meta-theme ‘A chance to do it right’ was illustrated through three main themes: ‘lasting losses’, ‘creating valuable moments’ and ‘best possible care in the worst imaginable’.
Camacho-Ávila et al.^26^ 2019 Spain	To provide a description of and understand the parents’ experiences of perinatal death.	Qualitative design	13 mothers 8 fathers	In-depth interview Inductive analysis Hermeneutic phenomenology	Eight sub-themes were grouped into three main themes: 1) Sensing a threat and anticipating the baby’s death: something is wrong in my pregnancy; 2) Emotionally draining: the shock of losing a baby and the suffering of giving birth to a dead child; and 3) We’ve had a baby: the need to legitimize grief and give the child an identity.

Continuing with phase four ‘Determining how the studies are related’, we identified an index article^20^ characterized by rich data and acceptable methodological quality and compiled a matrix (Supplementary file Table 3) that served as the basis for our synthesis. We noted first-order constructs (participant quotes) and second-order constructs (authors’ interpretations) from the Results sections in the included studies, and processed them into the matrix, listing studies horizontally and interpretative metaphors vertically, and maintaining the terminology used by the authors. By the end of phase four, we noted that the findings were comparable. All authors (CHP, THS, BD) were involved in the process of data extraction. In a meta-ethnography, the synthesis of findings is carried out through a process referred to as ‘translation’. The process entails identifying findings from one study and comparing them to those in another study, even if they are expressed in a different way.

In phase five, ‘Translating the studies into one another’, findings from the included studies were translated into each other. According to Noblit and Hare^13^ the findings can be comparable (reciprocal), they can stand in opposition to each other (refutational), or they can say something about the whole based on a set of parts (line of argument). The translation process confirmed our preliminary understanding that the data were mainly reciprocal. Thus, we translated the findings into one another by analogue translation to form new third-order concepts (our interpretation of the findings). This process was iterative and involved moving back and forth in the data.

### Synthesis process

During phase six, ‘Synthesizing translations’, four themes emerged, resulting in a line-of-argument synthesis and the development of an overarching metaphor. The process was interpretive yet systematic and we assessed and discussed our interpretations before reaching consensus. All authors were involved in the synthesis process. Finally, phase seven, ‘Expressing the synthesis’, gave an in-depth understanding of parents’ experiences with fetal death and the care they received between fetal death diagnosis and birth induction. This will be presented in the Discussion section.

### Ethical considerations

When conducting a meta-ethnography, data from peer-reviewed scientific primary research are used. Thus, it was not necessary to obtain ethical approval before conducting the study. However, we checked that all the primary articles had received ethical approval prior to implementation of the studies.

## RESULTS

We screened 329 articles for relevance and examined 43 articles in full text. Due to unsuitable methodologies and perspectives, as well as a lack of relevance and inadequate findings, we excluded 36 articles. Finally, 6 articles from the literature search and 1 article located through backtracking from the articles’ reference lists were included. The updated literature search did not yield any additional articles. Regarding the quality assessment of the included articles, two articles answered 9 of 10 questions on the CASP checklist. The remaining 5 articles answered all 10 questions. All studies were therefore considered to be of sufficiently high quality to be included in the meta-ethnography.

The seven qualitative articles included in our study^11,21-26^ included data from a total of 631 participants from Norway, Sweden, Spain, and the UK, and focused on parents’ experiences of fetal death (Table 3). The studies were conducted between 2004 and 2019, and the samples included 594 women, 12 men, and 25 parents, with samples varying from 11 to 515 participants.

Twelve of the women were from Norway, while 562 women were from Sweden. The two Spanish articles included a total of 21 women and 12 men, while the UK study included 25 parents. The data were collected through online questionnaires, interviews, semi-structured interviews, and in-depth interviews ranging from 1 month to 52 years after the death occurred. Most of the included studies used thematic analysis, but some used phenomenology or grounded theory.

### The synthesis

**Figure 2:**
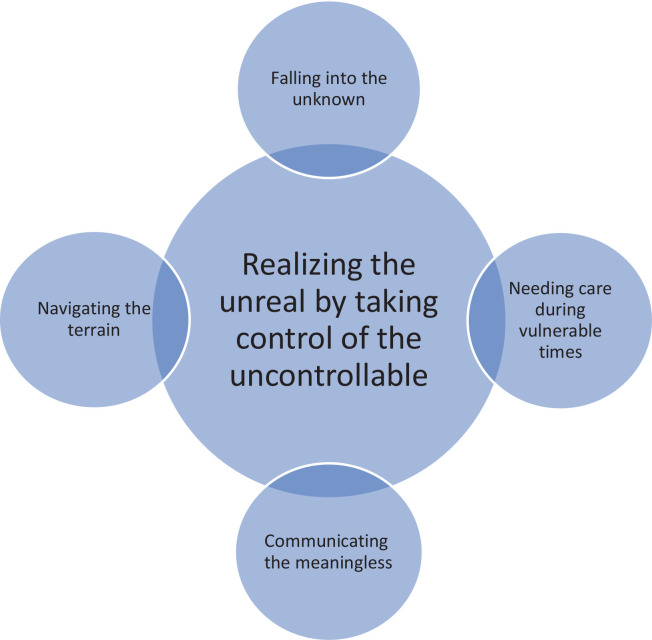
Synthesis

In the synthesis (Figure 2), ‘Realizing the unreal by taking control of the uncontrollable’ emerged as an overarching metaphor. The metaphor depicts an existential crisis where parents find themselves ‘falling into the unknown’ after having received information about the death. In this scenario, it was crucial to cope with and control a situation that seemed surreal. Some women portrayed the duration from diagnosis to birth induction as a phase of complete silence, leading to feelings of unreality and chaos. Their perception of time altered and their surroundings appeared non-existent. To prevent a breakdown, they required empathetic care, from HCPs who recognized the existence of their infant, which would assist them in managing their life situation. Eventually, they needed to discover methods to remain focused and prepare for the birth. In this context, sharing feelings of despair, grief, and anger with family and HCPs was of utmost importance. The metaphor was elucidated (Figure 2) through four main themes: 1) Falling into the unknown^11,21-24,26^; 2) Needing care during vulnerable times^11,21-26^; 3) Communicating the meaningless^11,21-26^; and 4) Navigating the terrain^11,21-25^. These are now described in detail.

### Falling into the unknown

The time between diagnosis and birth induction was described as chaotic^11,21-24,26^ and was regarded as the worst part of the process. Words such as ‘torture’ and ‘everything felt dark’ were used to describe the emotions they were experiencing, and one woman was so desperate that she considered taking her own life^11^. They experienced a world of chaos and loneliness when they realized that their child was dead^23,26^; everything fell apart and panic took over^21,23^:

*‘Everything just collapsed; bang! It was like everything just fell apart. My first thought was, just get the knife out and cut me open.’* ^23^

*‘The panic attack started. It can’t be true, I just screamed, it’s not true! I really screamed, I did, I screamed like, do something, help me, do something, I was in shock …^’^* ^23^

Even though some women were with their partner when they received the news, they still felt lonely and alone^23,26^ and in retrospect, they were unable to remember conversations they had had with midwives during this time^23^. As soon as the diagnosis was made, emotions flooded, and a long and difficult grieving process started^21,26^. At the beginning of the process, the women experienced strong feelings of disbelief, emptiness^11,21,22,26^, excruciating pain, outrage at having lost their child^26^, and anger^11,21,26^:

*‘… I was really cold … it was like I had no feelings … I was completely empty inside …’* ^21^

They did not understand what had happened or the significance of it; they felt helpless, in panic, and in shock. The news that their child had died was overwhelming, and the psychological pain was intense^23^. For many women, it was a hazy period marked by crying, darkness, despair, denial, and apathy^11^. They screamed, felt anxious, experienced panic attacks^11,23^, and vomited^23^. One woman threw the baby’s clothes against the wall and could not be left alone for fear of harming herself^11^. The strong emotions made it difficult to consider bodily needs, such as food intake, sleep, and preparation for childbirth^11^. One woman described the situation when she received the message as a nightmare. For another woman, the idea of giving birth to a dead child was unthinkable and impossible, and she likened it to an execution:

*‘… the nightmare begins when they tell you that your child is dead, the nightmare continues … but the real nightmare is when you begin to be aware of everything you have experienced …’* ^22^

After receiving a diagnosis, some women experienced anxiety and fear of death from being pregnant^11^. The child no longer appeared as their child but had become something unknown, distant, and frightening^21,24^, having invaded their bodies^24^. Some women responded by wanting to get rid of the dead child immediately^21,23,24^, while others asked for help to get it back^23^. They felt that they lost contact with the child and suffered from the absence of movement, the notion of having a live child, and the knowledge that this was no longer the case^11^. The expectations they had once had of the child were no longer there^26^. They had prepared for motherhood and were totally unprepared for death. Suddenly, the close bond between them and the child was gone^23^, and their expectations were broken^11,23,24,26^. The time from diagnosis to birth induction was experienced as a world of total silence where they felt like they fell ‘into the unknown’^23^. The situation felt unreal^21,23,24^ and they felt emotionally numb^23^:

*‘You don’t really understand what they’ve told you, even though you know the worst has happened. It’s unreal … that whole time … I don’t know … it’s like I can’t remember it, can’t describe my feelings. It was like being in a straitjacket.’* ^21^

### Needing care during vulnerable times

Several women experienced that their partners were not allowed to participate during the ultrasound examination^26^. Receiving the news that the child had died without a partner being present felt wrong. When the news that the child had died was communicated, they felt that their life situation suddenly changed completely and that they did not want to be alone^11,24,26^. Nevertheless, several women felt abandoned after the message had been conveyed^23^, experiencing that the HCPs left the room when they needed them the most^23,24^:

*‘… The gynecologist should have waited until my husband was there … she started to check me with the sonogram, looked at me and said: “No, I’m sorry, she doesn’t have any vital signs, she’s dead”. Just like that.’* ^26^

Several women felt lonely and neglected^22,23^, and one woman felt that she was given low priority because she carried a dead child in her womb^21^. Many felt abandoned when they did not receive the care and recognition they needed^11,21,26^. Parents found that doctors and midwives were unable to understand their state of mind or their needs. This caused intense frustration and sometimes angry and aggressive behavior^25^. For some parents, the care and support they received around the time of diagnosis was perceived as poorly managed and rude^22^:

*‘They exclude you, they distance you, they make you invisible …’* ^22^

The parents were told that they should have a new child quickly or that it was better to lose the child before than after birth. Attempts at well-intentioned words often had the opposite effect and made the parents feel misunderstood. They felt that their own sense of loss and grief was undermined, and this led to a denial of grief. Parents often felt that they received limited support from HCPs^22^, but when doctors and midwives spent time with them, this was perceived as supportive^11,25^. The emotional and interpersonal skills of HCPs affect parents’ experiences^21,22,25,26^, and thus, when midwives demonstrated empathy, respect and care, this was appreciated by the parents:

*‘The way she [midwife] spoke to me … I mean my mum was there, but it was like having another mum.’* ^25^

The parents experienced that some healthcare providers spent time with them, providing care and demonstrating that they were also affected by the situation. This acknowledgement of the child’s existence was important to the parents and helped them regain a kind of joy and pride that they were deprived of at the time of the diagnosis^25^. The participants described that midwives had the best ability to care^22^, and the importance of personal treatment was emphasized by one parent^26^:

*‘I was impressed by the delivery room midwife … the warmth with which she treated my wife. She let me stay with her, she held her hand and spoke to her gently … at such a hard time, that sort of personal treatment was comforting, and even today we still remember it as the most positive thing about that sad experience.’* ^26^

### Communicating the meaningless

The diagnosis was made using ultrasound^11,21-26^. During the examination, parents experienced that communication took place between the HCPs – both verbally and nonverbally – but did not convey their understanding of the situation with the parents^21,23^. The parents were sensitive to this verbal and nonverbal communication^25,26^, and the body language communicated by HCPs in the situation showed them that something was wrong with the child^21,23,26^. The parents had difficulty interpreting what they saw on the ultrasound screen. They feared that the child was sick or dead, but they still hoped for a miracle. They observed that the child was silent, without a heartbeat^23^, and they experienced that everyone in the room had full concentration on the screen^21,23^. The silence in the room increased the parents’ concern^21,23,26^, provided much information^26^, and confirmed their fears^23^:

*‘… everybody was totally silent … we asked, “What’s going on, what’s happening?”. They didn’t say anything … first there was a midwife, then a doctor came in, and then a gynecologist … they were all talking to each other but not to us …’* ^21^

This resulted in increased waiting times if the doctor who performed the ultrasound examination had to consult a colleague before informing the parents^23,26^. Some doctors delegated the task of communicating the message to a colleague. As a result, the women felt that the doctor did not take responsibility for communicating the bad news^23^. Often, information was unclear and poorly communicated^22,23,25^, and the parents questioned the HCPs’ clinical judgment and skills^25^. They needed causal explanations for the child’s death^11^, but they found that it was difficult to understand the information they were given. Nevertheless, some of the explanations led to a greater understanding of the cause of the child’s death^23^. Silence, a lack of explanation, and delays in providing information exacerbated parents’ suffering^22,23,26^, and this triggered anxiety^23,26^, tension, uncertainty^26^, and a feeling that the system had failed them^22^. Thus, caring nonverbal language was crucial for the parents when they received the news^26^.

The parents preferred encountering experienced HCPs with good communication skills^22^ and who were calm and reliable, and had sufficient time to share information about what was going to happen^21^ which the parents did not know^25^. Some parents were unable to handle the information they received, maintaining an emotional distance from the situation and their surroundings^25^. They hardly experienced that they were involved in decision making regarding their situation^11,22,24^; instead, HCPs presented suggestions that they believed to be the best for them, and the parents approved this^24^. Some parents even felt that they were pushed to make decisions when they felt they were not mentally capable of doing so^25^. They found themselves in a ‘nightmare’^22^ and were unable to make decisions^22,24^. Only occasionally did parents feel that they were given sufficient space to adapt to the situation^22^:

*‘Either they help you or they orient you or you cannot make any decision for yourself …’* ^22^

### Navigating the terrain

The parents felt that they found themselves facing ‘the unknown’^22,23^. They needed help with the situation^22,23^ as well as professional guidance and information^22,25^. They experienced being in an unreal situation from which they needed to flee^21^; they denied death and searched for confirmation of its absence, hoping that a medical misjudgment had been made^22^. Physical proximity and conversations with friends, family, and HCPs helped them stay focused on the situation. For many, sharing feelings of despair, grief, and anger helped them structure and understand the situation more easily. Others chose to address the situation by avoiding people who could have supported them^11^ or found it difficult to inform their families about the situation^11^.

After the parents had received the news that the child had died, the women were often allowed to choose whether they wanted to stay in the hospital overnight or go home and return to the hospital the next day. Often, they were advised to wait one or two days before starting the induction^21,24^. Influencing the outcome of their birth was impossible. Thus, it became important for them to influence the choice of time for the induction of labor and where to spend their time awaiting the induction. The waiting time was frustrating^25^, meaningless, and involuntary, and some wanted the pregnancy to end as soon as possible^24^. The women used the time leading up to birth induction to make the situation comprehensible. They sought information and planned the birth and the aftermath with their loved ones. Some families stayed together, lighting candles, talking to the baby in the womb, and looking at ultrasound images. Women took pictures of their stomach, or they gave the baby a name. For others, it was essential to contact a priest and start planning the funeral^11^ or get rid of the clothes they had bought for the baby^24^. Others packed a bag for birth, thinking, praying, and crying in solitude^11^:

*‘The evening was spent packing, crying, and trying to fix a babysitter in time for the induction the next day.’* ^11^

They described having feelings of no longer being in contact with the outside world and that everything ceased to exist. Their sense of time changed. Eventually, they managed to gain some control over the situation^11^. When labor was induced, the women felt that they had accepted a normal birth and had to rely on recommendations that vaginal delivery was the best option for them for both physical and mental reasons^21^. This information led the women to change their idea of not seeing meaning in giving birth to a dead child to accepting and then carrying out the birth^11,21^. Despite their anxiety and pain, they felt they handled the situation^24^:

*‘I focused on getting it over and done with, I was mostly thinking about putting it behind me. I did not mourn for the baby … I just thought that we need to get this done …’* ^24^

Some women focused on the future and the possibility of having a new child, and several participants said they would eventually cope with the situation even though it seemed impossible. The worst possible thing had happened, but they had managed to face difficult situations before and knew they would be able to face this situation^24^.

## DISCUSSION

The analysis of 7 qualitative studies exploring parents’ experiences of fetal death and the care they received between diagnosis and birth induction resulted in the overarching metaphor

‘Realizing the unreal by taking control of the uncontrollable’. This metaphor encapsulates an existential crisis; parents characterized the time from the diagnosis of fetal death to birth induction as surreal and chaotic. In their quest to remain focused and prepare for the birth, they found empathetic care to be crucial. Our study underscores the importance of midwives demonstrating bravery in confronting the parents’ distress and providing compassionate care to mitigate their suffering.

Parents who experience fetal death go through an extremely sensitive time between diagnosis and birth induction. Their suffering is evident, with strong descriptions related to the need for care and support during the crisis^11,21-26^. The concept of suffering is central to the work of Eriksson^27^ that suffering can be regarded as an ontological concept, a deep and vital condition of life. Suffering may be experienced as evil and meaningless. Nevertheless, through lived experiences of suffering, it is possible to ascribe meaning to suffering. Eriksson^27^ refers to the caring encounter as a drama of suffering consisting of three acts: 1) the confirmation of suffering, 2) the suffering itself; and 3) reconciliation. Confirming a person’s suffering implies mediating to the other that ‘I see’ the person’s suffering. According to Eriksson^27^, confirmation of suffering can happen through a look, a touch, or by use of words, signaling that you are willing to be there for the other person and share their suffering. Compassion alleviates suffering, and true caring encounters take place where suffering and compassion meet^27^.

Severe crises, such as experiencing fetal death, may entail experiences of abandonment, loneliness, worthlessness, and a sense of chaos, resulting in anxiety and a frantic search for meaning^28^. We found that when parents received confirmation that the baby had died, it led to severe mental stress and immediate feelings of loneliness^11,21-24,26^. In this phase, the deepest feeling of loneliness and the greatest suffering was the experience of not being seen by others^27^. Our findings demonstrate situations where parents experienced not being seen by midwives or HCPs^11,21-23,25,26^. Some even perceived HCPs to be indifferent, hostile, and unable to perceive their state of mind and needs^25^. The disorder that manifests among parents in the period between the diagnosis of fetal death and birth induction requires immediate emotional involvement from HCPs^8^ and can be compared to Eriksson’s ‘Drama of suffering’^27^, where HCPs must assume the important role of being a co-actor in helping to alleviate suffering, supporting the parents, and helping them freely express their feelings of pain, grief, guilt, and anger.

The purpose of care is to alleviate suffering, and the parents’ experience of care was influenced by how the midwife or HCP met and supported the suffering parents^29,30^. The parents appreciated midwives who offered to stay in the room^31^, while the feeling of loneliness intensified the suffering if HCPs left the room after the news had been communicated^8^. It was important that the midwife had the desire and the courage to face the parents’ suffering and alleviate it, not only by talking, giving of herself, comforting, encouraging, supporting, and conveying hope, but also by sharing hopelessness^27^. She must dare to stand with the parents and be close to them during the crisis even though it may be difficult and challenging^27^. Due to the low incidence of fetal deaths compared to live births, midwives and other HCPs do not receive much training in providing care to these parents^9^. Nevertheless, the way the diagnosis is communicated will be remembered by the parents and emphasizes the importance of training in empathetic communication skills^7^. Fetal death was occasionally regarded as an unexpected clinical tragedy, and some parents felt that HCPs downplayed the significance of the child’s death with comments that they already had a child, or the assurance that they could have another one later^8^. Such well-intentioned comments often had the opposite effect^8^ and were instead hurtful. The parents did not experience losing a fetus, they experienced losing a child, a child they greatly wanted.

Research has demonstrated that HCPs, as well as midwives, lack theoretical and practical knowledge about fetal death^7,10,12,31,32^, as well as knowledge related to communication skills and caring in encounters with parents who experience fetal death^22,33^. The lack of training in caring for suffering parents may hamper the ability to provide meaningful support, which in turn may be the main reason why midwives and other HCPs experience this situation as unsafe, stressful and emotionally challenging. In such situations there is a risk that midwives choose ‘task-oriented-care’ over proximity^34,35^. Ensuring that all HCPs receive quality support and training is therefore essential for the guidance and improvement of maternity care^36^.

Our findings demonstrate that communication is, in many ways, both fundamental and crucial for parents’ experience of care^11,21-26^. The way doctors communicate a diagnosis has an impact on how parents perceive care and support at the time of diagnosis^31,37,38^. According to Pullen et al.^38^, the absence of empathy during the ultrasound examination can contribute to negative experiences. Parents have a great need for care and information during the ultrasound, at the time of confirmation of fetal death, and in the period after^11,21-26^. Thus, the importance of HCPs trying to create a relationship of trust is essential before communicating the bad news in a considerate manner where parents feel that they are allowed to react^37^.

According to Kwame and Petrucka^39^, communication is seen as a core component of care and has a significant impact on interactions between parents and midwives. Communication becomes therapeutic if it is centered around the parents, thus promoting trust and mutual respect in the caring process^39^. We found that when midwives communicated well and showed empathy, warmth, and respect, it was appreciated by the parents^11,21-26^. Dismissive, blunt, cold, or inconsiderate communication was perceived as hurtful^31^ and contributed to increasing the parents’ burden^8^. Sensitivity, a calm touch, reassurance, and small comforting gestures such as hugs or a hand to hold were highly valued and contributed to the parents experiencing support^31,38^. It is important to consider that in the caring encounter, we communicate in everything we say and do, even when we do not say or do anything^40,41^. The parents’ experiences of nonverbal communication were elucidated in our findings^21,23,25,26^, confirming that, if the midwife is present and is demonstrating consistency between her verbal and nonverbal communication, the message will be understood by the parents as genuine, reliable, and trustworthy. Care is communicated through verbal as well as nonverbal communication, and its quality depends on the HCPs’ communication skills and interpersonal relationships. Care and communication are thus in continuous interaction; caring does not exist without communication, and communication does not exist without caring. Thus, if parents are helped to realize the unreal through optimal care performance where communication is central, they will be able to more easily achieve control and mastery over the uncontrollable situation they find themselves in.

Our findings demonstrate the tough waiting time from the diagnosis of fetal death to birth induction^11,21-25^. Thoughts of guilt, preparations for childbirth, need for support, one’s own coping, and attempts to regain control on the way to reconciliation were all described.

If the parents feel that they receive the individual support, guidance, and genuine care they need, they will be better equipped to experience coping and reconciliation. They will more easily be able to mourn the loss of the child without also having to mourn the lack of care and lack of communication they experience in their encounters with HCPs^27^. Parents may find themselves in chaos and shock, fearing that life will never be the same. Nevertheless, if their suffering is confirmed and they receive genuine care and communication, this may contribute to a less painful grieving process. Midwives and other HCPs are supposed to help the parents in finding an opportunity to experience reconciliation. However, if the midwife is unable to be a co-actor in the drama, if she is unable or unwilling to help alleviate the parents’ suffering, the parents may experience increased suffering. This may result in parents not reaching the third part of the drama of suffering, which is reconciliation^27^. Midwives who provide time and space for the outlet of parents’ emotions are therefore important^27^. This is supported by Eriksson^27^, who states that the sufferer needs time and space to suffer, and if one explains it away or quickly tries to find a cause, parents may be deprived of the opportunity to suffer. According to Eriksson^27^, grief for the lost is found in suffering, and through suffering the parents will change and have the opportunity for reconciliation. Only when they come to terms with the situation can meaning in the suffering arise and opportunities come to light. Suffering can then be transformed by insight into new possibilities and thus awaken a glimmer of hope^27^.

### Strengths and limitations

This study synthesizes parents’ experiences with the care they received in the period between the diagnosis of fetal death and birth induction. Systematic literature searches resulted in more than 300 articles being screened for relevance. However, only seven articles fulfilled the inclusion criteria. This demonstrates that there is a lack of research illuminating the challenging period between receiving a stillbirth diagnosis and inducing labor. According to Noblit and Hare^13^, meta-ethnography can be carried out with two to four articles, while other researchers argue that meta-ethnography should include ten or more articles^19^. We considered it sufficient to include seven articles due to the narrow topic of the study, the small number of informants, and the availability of rich data.

We included articles published in the last two decades, and in retrospect we found that this did not seem to have any bearing on the findings. Our choice of keywords, the inclusion and exclusion criteria, and the choice of databases may have influenced the literature search, resulting in the loss of relevant studies. However, our sample comprised 631 participants from Norway, Sweden, Spain and the UK, describing a variety of challenges related to the short timespan between diagnosis and birth induction. Thus, we found that this study provided rich data to support the research aim. Due to cultural and social differences in maternity care between Western and non-Western countries, only articles from Western countries were included. Nevertheless, non-Western articles could have contributed more nuances to the findings and thus contributed important knowledge to the study.

We are aware that the authors’ backgrounds may have influenced the analysis. We have broad clinical experience in nursing and maternity care (nursing and midwifery), including encountering families who have experienced losing their infants during pregnancy or birth. The research experience of the authors spans a range of qualitative methods, including the use of meta-ethnography. This approach enabled a reflexive interdisciplinary analysis.

## CONCLUSIONS

The challenging reality that their infant had died plunged the parents into emotional turmoil. They were in desperate need of assistance and support to manage the tragic situation; however, they felt neglected and experienced a communication gap with the healthcare providers. This further increased their distress. Sensitive communication, clear and understandable information, shared decision-making, and respect for individualized care are useful strategies for healthcare providers in their encounters with parents. It is of great importance that midwives and doctors receive good training prior to encounters with parents who experience fetal death, in order to provide good care and support for parents throughout the process.

## Supplementary Material



## Data Availability

The data supporting this research can be found in the Supplementary file.
