# Supplementary material for: A meta-ethnography exploring parents’ experiences of fetal death and the care they received between diagnosis and birth induction
Source: Eur J Midwifery. 2025 Mar 19;9:10.18332/ejm/200614. doi: 10.18332/ejm/200614 (PMC11917500; doi:10.18332/ejm/200614)
Supplement: Supplementary file 1 [file EJM-9-16-s1.pdf]

Supplementary Table 1. The eMERGe meta-ethnography reporting guidance*

Phase	Criteria	Page
Selecting meta-ethnography and getting started	**1. Rationale and context for the meta-ethnography**<br>Describe the gap in research or knowledge to be filled by the meta-ethnography, and the wider context of the meta-ethnography	1–2
	**2. Aim(s) of the meta-ethnography**<br>Describe the meta-ethnography aim(s)	2
	**3. Focus of the meta-ethnography**<br>Describe the meta-ethnography review question(s) (or objectives)	2
	**4. Rationale for using meta-ethnography**<br>Explain why meta-ethnography was considered the most appropriate qualitative synthesis methodology	2
Deciding what is relevant	**5. Search strategy**<br>Describe the rationale for the literature search strategy	3
	**6. Search processes**<br>Describe how the literature searching was carried out and by whom	3-4
	**7. Selecting primary studies**<br>Describe the process of study screening and selection, and who was involved	3–4
	**8. Outcome of study selection**<br>Describe the results of study searches and screening	3–4
Reading included studies	**9. Reading and data extraction approach**<br>Describe the reading and data extraction method and processes	4–5
	**10. Presenting characteristics of included studies**<br>Describe characteristics of the included studies	5
Determining how studies are related	**11. Process for determining how studies are related**<br>Describe the methods and processes for determining how the included studies are related:<br>- Which aspects of studies were compared AND<br>- How the studies were compared	4–5

	**12. Outcome of relating studies**<br>Describe how studies relate to each other	4–5
Translating studies into one another	**13. Process of translating studies**<br>Describe the methods of translation:<br>- Describe steps taken to preserve the context and meaning of the relationships between concepts within and across studies<br>- Describe how the reciprocal and refutational translations were conducted<br>- Describe how potential alternative interpretations or explanations were considered in the translations	4–5
	**14. Outcome of translation**<br>Describe the interpretive findings of the translation	4–5
Synthesizing translations	**15. Synthesis process**<br>Describe the methods used to develop overarching concepts (“synthesized translations”)<br>Describe how potential alternative interpretations or explanations were considered in the synthesis	4–5
	**16. Outcome of synthesis process**<br>Describe the new theory, conceptual framework, model, configuration, or interpretation of data developed from the synthesis	5–12
Expressing the synthesis	**17. Summary of findings**<br>Summarize the main interpretive findings of the translation and synthesis and compare them to existing literature	12–15
	**18. Strengths, limitations, and reflexivity**<br>Reflect on and describe the strengths and limitations of the synthesis:<br>- Methodological aspects - for example, describe how the synthesis findings were influenced by the nature of the included studies and how the meta-ethnography was conducted<br>- Reflexivity - for example, the impact of the research team on the synthesis findings	15-16

	**19. Recommendations and conclusions**<br>Describe the implications of the synthesis	16

*France EF, Cunningham M, Ring N, et al. Improving reporting of meta-ethnography: The eMERGe reporting guidance. Psychooncology. 2019;28(3):447-458. doi:10.1002/pon.4915

Supplementary Table 2: Search terms

	SCOPING SEARCH	COMPREHENSIVE AND UPDATED SEARCH
S	Mothers OR<br>Parents OR<br>Midwife*	Parents OR Mothers OR Midwifery OR Midwifery service* OR Midwifery care
PI	Stillbirth OR<br>IUFD OR<br>Fetal death OR<br>Intrauterine death	Stillbirth OR Still birth OR Stillborn OR Still born OR Fetal death OR Perinatal death OR Dead baby in utero OR Neonatal death OR Perinatal loss OR Pregnancy loss OR IUFD OR Intrauterine death OR Death in utero
D	Interview	Interview OR In-depth interview OR Focus group OR Group interview OR Narratives OR Questionnaires OR Diary OR Audio-diaries OR Descriptive OR Phenomenolog* OR Hermeneutic* OR Grounded theory
E	Experience OR<br>Bereave*	Parental experience* OR Perception* OR Views OR Perspective* OR Parental attitudes OR Maternal attitudes OR/experiences Parental attitudes OR/experiences Bereavement OR Grief OR Lived experience
R	Qualitative	Qualitative research OR<br>Qualitative study OR<br>Qualitative methods

Supplementary Table 3: Matrix for analysis and synthesis

Theme 1: Falling into the unknown						
Erlandsson, K., *et al.*	Trulsson, O. & Rådestad, I.	Martínez-Serrano, P., *et al.*	Rådestad, I., *et al.*	Malm, M. C., *et al.*	Downe, S., *et al.*	Camacho-Ávila, M., *et al.*
2011	2004	2019	2014	2011	2013	2019
Some mothers described themselves as being in a state of chaos during the time between the diagnosis of the intrauterine death and the induction. To them, that time was the worst part of the whole process.<br>When looking back at that time, memories are hazy. Some mothers described emotional turns of emptiness, darkness, chaos, despair, denial, or a sense of unreality and blunted feelings. When mothers were occupied with their emotions, they could not focus and sometimes their bodies reacted. They could not eat or sleep, which meant that they did not prepare themselves for the pending birth.<br>Some respondents described anxiety of such severity that they could not be left alone in a room even for a short period. They considered their frailty as a threat to themselves and feared that they might harm themselves. Others, in despair, threw baby things around at home, screaming and crying, getting mad.<br><br>Some mothers experienced mortal dread and fear of death when they were pregnant with a dead baby.<br><br>Some mothers suffered from the absence of signs of a living baby in utero, with ambiguous feelings vis-à-vis their bellies. Being disconnected from the baby sometimes also meant	They wanted to get rid of the dead child immediately.<br><br>The dead baby within was perceived as indefinable and frightening.<br><br>They were in turmoil with feelings of anger and sorrow.<br><br>The women described the time immediately after they learned their baby was dead as unreal and numbing.<br><br>They had difficulty comprehending what had happened and what it all meant.	An initial reaction of unreality was common among all parents that they described in diverse ways. The unreality was accompanied with disbelief and attempts to flee from the situation,	Some screamed and some had physical reactions such as vomiting; others asked for a cesarean section. Some said that they pleaded for help feeling that 'I cannot do this,' 'Please do something shake her bring her back to life,' or 'Cut me open.' The mothers described panic attacks occurring at the moment they understood that the baby was dead.<br><br>They described the time immediately after they understood their baby was dead as being unreal and crying out for help and had difficulty comprehending what had happened and what it all meant.<br><br>The mothers said that they had entered a world of chaos and loneliness – in total silence.<br><br>The mothers reacted to the death with feelings of unreality, panic and shock. The devastating understanding that their baby was dead was overwhelming and the mothers experienced an intense psychological pain. At that particular moment the mothers reported experiencing a sense of 'free falling' into something unknown.<br><br>In the moment of the confirmation that their baby was dead, the mothers	Some of the mothers talked about the baby as something strange that had been taking possession of their body. Giving birth to a dead baby was an unimaginable and bizarre thought for several mothers. They described it as impossible, and one mother even associated it with an execution<br><br>The mothers described a sense of unreality that characterized the time before induction of labor.<br><br>The insight of being the mother to a child who no longer exists came to the mothers in this period. They described it as having to face broken expectations but not being given anything instead.		The confirmation of a baby's death is the start of a long, hard journey that makes up part of the parents' grief process. The emotional outpouring is produced once the death has been confirmed.<br><br>Parents recalled that they felt disbelief, anguish, anger, emptiness, insurmountable pain, and outrage about the loss of their baby. This represents the first step in working through their grief.<br><br>The pain of the loss increases due to uncertainty or loneliness.<br><br>Even when surrounded by many people, mothers sometimes felt just as lonely as they did when they were physically alone.<br><br>The expectations the parents had for their baby are no longer present.

imagining a living baby soon to be born, despite the knowledge that it could not be.			experienced loneliness, although they were in the company of the baby's father or another companion.<br><br>The mothers did not remember any dialogue taking place between them and the clinician or other staff at that moment.<br><br>The death was unexpected in a sense because up until that moment the women were prepared for motherhood and then, in an instant, the close tie between the mother and the baby could be described as having been abruptly cut off.			

© 2025 Pettersson C.H. et al.
